# Fast and slow skeletal myosin binding protein-C and aging

**DOI:** 10.1007/s11357-022-00689-y

**Published:** 2022-11-21

**Authors:** L. R. Perazza, G. Wei, L. V. Thompson

**Affiliations:** Department of Physical Therapy, College of Health & Rehabilitation Sciences: Sargent College, Boston University, 635 Commonwealth Ave, Boston, MA 02215 USA

**Keywords:** Aging, Muscle contractility, Phosphorylation

## Abstract

Aging is associated with skeletal muscle strength decline and cardiac diastolic dysfunction. The structural arrangements of the sarcomeric proteins, such as myosin binding protein-C (MyBP-C) are shown to be pivotal in the pathogenesis of diastolic dysfunction. Yet, the role of fast (fMyBP-C) and slow (sMyBP-C) skeletal muscle MyBP-C remains to be elucidated. Herein, we aimed to characterize MyBP-C and its paralogs in the fast tibialis anterior (TA) muscle from adult and old mice. Immunoreactivity preparations showed that the relative abundance of the fMyBP-C paralog was greater in the TA of both adult and old, but no differences were noted between groups. We further found that the expression level of cardiac myosin binding protein-C (cMyBP-C), an important modulator of cardiac output, was lowered by age. Standard SDS-PAGE along with Pro-Q Diamond phosphoprotein staining did not identify age-related changes in phosphorylated MyBP-C proteins from TA and cardiac muscles; however, it revealed that MyBP-C paralogs in fast skeletal and cardiac muscle were highly phosphorylated. Mass spectrometry further identified glycogen phosphorylase, desmin, actin, troponin T, and myosin regulatory light chain 2 as phosphorylated myofilament proteins in both ages. MyBP-C protein-bound carbonyls were determined using anti-DNP immunostaining and found the carbonyl level of fMyBP-C, sMyBP-C, and cMyBP-C to be similar between old and adult animals. In summary, our data showed some differences regarding the MyBP-C paralog expression and identified an age-related reduction of cMyBP-C expression. Future studies are needed to elucidate which are the age-driven post-translational modifications in the MyBP-C paralogs.

## Introduction

Aging is strongly associated with a decline in muscle contractility, affecting especially skeletal muscle strength and cardiac diastolic function [1–4]. Muscle contractility, in both skeletal and cardiac muscles, is dependent on the structural arrangements of the sarcomeric proteins and their combined activities. To date, studies probing the cellular functions of myosin binding protein-C (MyBP-C) enhance the understanding of the complex pathogenesis associated with diastolic dysfunction leading to cardiac disease [5]. More recently, there is emerging evidence suggesting MyBP-C proteins may contribute to pathologies associated with skeletal muscle diseases, such as muscular dystrophy and arthrogryposis myopathy [6–10]. Yet, the role of MyBP-C in fast skeletal muscle dysfunction with aging remains unknown.

MyBP-C is a multidomain protein found in the C region of A bands of the sarcomere. There are three distinct paralogs (isoforms), including the cardiac (c), fast skeletal (f), and slow skeletal (s) MyBP-C proteins encoded by three separate genes (MYBPC3, MYBPC2, and MYBPC1), respectively [8, 11]. These paralogs play unique muscle-specific structural and regulatory roles in myosin-actin interactions and contractility based on detailed, in vitro biophysical investigations [6, 8]. Importantly, isoform-specific sarcomeric proteins, such as myosin heavy chain and troponin, show marked changes in composition with aging, which have functional significance [12–15]. Yet, it is unknown whether the expression of the skeletal muscle MyBP-C paralogs (slow and fast skeletal MyBP-C) changes in the fast tibialis anterior (TA) muscle with aging.

The state of post-translational modifications of sarcomeric proteins is critical to the regulation of contractility. In particular, phosphorylation of cMyBP-C is a mediator of diastolic function such that a high level of phosphorylation is essential to normal cardiac function, whereas hypophosphorylation is associated with heart failure [16–18]. With aging, there is evidence that changes in the phosphorylation status of specific residues in cMyBP-C may be a major contributor to the age-related loss of diastolic function in murine hearts [19]. Although the MyBP-C paralogs have yet to be fully described in aging skeletal muscles, there is differential phosphorylation of specific residues in the sMyBP-C paralog within the slow soleus and the fast flexor digitorum brevis muscles early in the lifespan (between the ages of 2 and 14 months) from WT mice and from mice with dystrophy (*mdx* mouse model) providing sufficient rationale for further investigation of this sarcomeric protein and its paralogs as potential factors contributing to the complex nature of sarcopenia [10, 20]. Skeletal and heart muscles are also rich in mitochondria and have high metabolic demands, which provide microenvironments whereby the sarcomeric proteins are highly susceptible to oxidative damage [3, 21, 22]. Therefore, understanding the post-translational oxidative state of skeletal muscle MyBP-C paralogs with age may offer a mechanism contributing to sarcopenia.

The overall goal of this study was to begin to characterize MyBP-C and its paralogs in the fast tibialis anterior muscle from adult and old mice. We tested the hypothesis that both fMyBP-C and sMyBP-C paralogs would be detected; however, the abundance of the fMyBP-C would be greater than the sMyBP-C in both age groups. According to age-related changes in protein isoform compositions within skeletal muscles, we hypothesized that the expression levels of the fMyBP-C paralog would decrease with age. Lastly, because post-translational modifications of proteins, such as phosphorylation and carbonylation, impact contractile function, we predict that the levels of phosphorylation of MyBP-C would be decreased with age, whereas carbonyl-bound to MyBP-C would increase. These hypotheses were addressed by making immunoreactivity measurements in the tibialis anterior muscles from adult and old mice, which provided a platform for addressing the specific MyBP-C paralogs present in skeletal muscles. We also investigated the cMyBP-C paralog from ventricular muscles to confirm previously published works.

## Materials and methods

### Animals and muscle tissue

Skeletal (fast tibialis anterior, TA) and cardiac (left ventricle) muscle tissues from adult and old C57Bl/6 male mice were harvested, quick-frozen in liquid nitrogen, and stored at – 80 °C. The tissues were randomly selected from a previous study that demonstrated significant age-related changes in behavioral performance (i.e., Rota-rod and inverted grip test) and in vitro muscle contractility (peak tetanic force in EDL and soleus muscles [23]). For TA muscle analyses, the adult (*n* = 8) and old (*n* = 8) mice were aged nearly 13 months and 29 months, respectively. For the cardiac analyses, the adult (*n* = 18) and old (*n* = 12) mice were aged nearly 13 months and 29 months, respectively. Animals were kept under a regular light/dark cycle and under an ad libitum regimen [23, 24]. All experimental procedures involving animals were conducted in accordance with the University of Minnesota’s Animal Care and Use Committee.

### Extraction of myofibrillar proteins

Myofibrillar proteins were extracted from the TA muscles as previously described [21]. Fractionation of muscle samples into extracts 1 and 2 (enriched for cytosolic and myofibrillar proteins, respectively) was performed on ice. Briefly, the TA muscles (24–41 mg) were diced, placed into 500 μl of subfractionation buffer 1 (20 mM imidazole and 5 mM EDTA, pH 7.4), containing 1:100 diluted halt protease and protease inhibitor cocktails (100x, Thermo Scientific), and immediately homogenized. Following a 15-min centrifugation at 12,000 g at 4 °C, the supernatant was collected, and the remaining pellet was homogenized with 250 μl of buffer 1, and centrifugation was repeated. The supernatants were combined, forming extract 1, and the remaining pellet was then homogenized in 250 μl of subfractionation buffer 2 (0.5% TFA, 1 mM tris (2-carboxyethyl phosphine) hydrochloride) followed by a 15-min centrifugation at 12,000 g. The supernatant was collected, and the remaining pellet was homogenized with 100 μl of buffer 2, and centrifugation was repeated. The combined supernatants comprised extract 2. In contrast to the TA muscles, the heart muscles were homogenized in ice-cold RIPA lysis buffer (Thermo Scientific) supplemented with halt protease & protease inhibitor cocktails (Thermo Scientific) and immediately homogenized. The muscle homogenates were centrifuged at 16,000 g, 20 min at 4 °C. Protein concentration of the homogenates (TA, heart) was determined using the bicinchoninic acid protein assay kit with BSA as the standard. The homogenate samples were aliquoted and stored at – 80 °C.

### Immunoblotting

Before immunoblotting, muscle homogenates were separated by SDS-PAGE using a 4–15% Mini-PROTEAN® TGX Stain-Free™ Protein Gel (12 well, 20 μl) at RT, 100 V for ~ 1 h. Gels were transferred to nitrocellulose membranes (0.2 μm, Bio-Rad) on a Mini Trans-Blot cell at 30 V overnight in a buffer containing 192-mM glycine, 25-mM Tris, 0.025% SDS, and 20% methanol. To determine total proteins within the TA muscle, the blots were stained with REVERT™ Total Protein Stain and Wash Solution Kit (LI-COR Biosciences) and imaged at 700 nm (red) with Odyssey imaging system (LI-COR Biosciences). Next, the stain was reversed, according to REVERT™ protocol, with reverse solution (0.1 M NaOH, 30% methanol in H_2_O). To determine total proteins within the heart muscle, the blots were stained with Ponceau S (red stain), rinsed, and imaged (700 nm, red). Next, the membrane was destained with 0.1 M NaOH. The blots (TA, heart) were blocked in Odyssey Blocking Buffer-TBS (LI-COR Biosciences) at RT for 1 h and then incubated with each corresponding primary antibody in TBST buffer containing 0.2% Tween 20 overnight at 4 °C (Table 1). After washing with TBST containing 0.1% Tween (5 × 5 min) the blots were probed with secondary antibody IRDye800CW (LI-COR Biosciences) goat anti-mouse for BF-35 (MHC) and cMyBP-C and goat anti-rabbit for sMyBP-C and fMyBP-C, then imaged at 800 nm (green) (Table 1, 2).

**Table 1:** Antibodies

		Primary antibody			Secondary antibody	
Target proteins	Protein load (µg)	Type	Host	Dilution	Goat anti-Host	Dilution
MHC (BF-35)	10	M	M	1:50	M	1:15,000
sMyBP-C	10	P	R	1:500	R	1:15,000
fMyBP-C	10	P	R	1:500	R	1:15,000
cMyBP-C	15	M	M	1:1000	M	1:15,000
cMyBP-C p(Ser282)	20	P	R	1:500	R	1:10,000
DNP	5	P	R	1:150	R	1:10,000

All antibodies were isotype IgG. Host species rabbit (R) or mouse (M); primary antibody sMyBP-C (slow type) was purchased from Sigma; fMyBP-C (fast type) from Abnova; cMyBP-C (cardiac) from Santa Cruz; cMyBP-C phosphorylated at Ser^282^ from Enzo; DNP (anti-DNP antibody) from Millipore; antibody BF-35 for MHC type I, 2A, and 2B from DSHB (Developmental Studies Hybridoma Bank). Secondary antibodies: IRDye800CW or IRDye680RD goat anti-rabbit (R) or mouse (M) were from LI-COR Biosciences

**Table 2:** Proteins identified by mass spectrometry

Sample (~ kDa)	Unique	Total	Reference	Gene symbol	MW (kDa)	Protein
1 (130)	80	412	sp|Q5XKE0|MYPC2_MOUSE	Mybpc2	127.27	Myosin binding protein C2
	64	125	tr|Q6P6L5|Q6P6L5_MOUSE	Mybpc1	126.12	Myosin binding protein C1
2 (100)	44	56	sp|Q9WUB3|PYGM_MOUSE	Pygm	97.22	Glycogen phosphorylase, muscle form
3 (50)	38	54	sp|P31001|DESM_MOUSE	Des	53.47	Desmin
4 (45)	34	243	sp|P60710|ACTB_MOUSE	Actb	41.71	Beta-actin
5 (24)	16	39	sp|Q9QZ47|TNNT3_MOUSE	Tnnt3	32.22	Fast skeletal muscle troponin T
6 (18)	31	206	sp|P97457|MLRS_MOUSE	Mylpf	18.94	Myosin regulatory light chain 2, skeletal

After staining with Sypro Ruby, 6 gel bands at MW of 130 to 18 kDa were sliced for mass spectrometry analyses

To quantify carbonylated protein content, OxyBlot protein oxidation detection kit (Millipore) was used according to the manufacturer’s protocol. Briefly, an aliquot of the muscle homogenate was denatured and derivatized using SDS and DNPH (2,4-dinitrophenylhydrazine) solution at RT for 15 min, then adding neutralization solution in 1.5-fold of the homogenate sample volume. An equal amount of protein (5 μg) of each sample was loaded, separated, transferred to a nitrocellulose membrane, stained, and imaged as outlined above. The membranes were blocked in 1%BSA-PBST buffer (containing 1% BSA and 0.05% Tween-20 in PBS) at RT for 1 h, then probed with a rabbit anti-DNP primary antibody diluted in 1%BSA-PBST for 1 h at RT on an orbital shaker. Next, the membranes were washed with PBST and then probed with goat anti-rabbit secondary antibody IRDye800CW diluted in 1%BSA-PBST for 1 h at RT with shaking. After washing as previously stated, the membranes were then treated, imaged, and analyzed.

Densitometry analysis was performed using Odyssey infrared imaging system application software version 3.0 (LI-COR Biosciences). Quantification was performed by total protein normalization methods with two equations: (1) lane normalization factor (LNF) = signal / signal for lane with highest signal; (2) normalized signal = target band signal / lane normalization factor. A TA muscle and a heart muscle were prepared as blot controls in order to compare samples across the blots. Final protein content of each individual sample was expressed as a ratio to the blot control (AU).

### Phosphorylation

Phosphorylation of TA and heart muscle myofibrillar proteins was estimated by Pro-Q® Diamond phosphoprotein staining method according to the manufacturer’s instructions. In brief, each sample aliquot (150 µl) was cleaned by methanol and chloroform. The proteins were separated by SDS-PAGE using a 4–15%, electrophoresed at RT, 80 V for 1.5 h and stained with Pro-Q® Diamond phosphoprotein gel stain (Molecular Probes). Subsequently, gels were stained with fluorescent Sypro Ruby stain (Molecular Probes) to estimate the amount of total and targeted proteins. The gels were scanned with ChemiDoc MP Imaging System (Bio-Rad), and images were analyzed by Image Lab 6.1 software to determine the density of each protein band: myosin binding protein-C (127 kDa), glycogen phosphorylase (GP, 97 kDa); desmin (53 kDa); actin (42 kDa); fast skeletal muscle troponin T (fTnT, 32 kDa), and myosin regulatory light chain 2 (MLC2, 19 kDa).

Because of the availability of the cMyBP-C-p(Ser282)-specific antibody, we also performed immunoblotting of the heart samples and probed with primary antibodies for both cMyBP-C and cMyBP-C-p(Ser282) (Table 1). In these experiments, the blots were probed with secondary antibody IRDye800CW goat anti-mouse for cMyBP-C and IRDye680RD goat anti-rabbit cMyBP-C-p(Ser282), then imaged at 700 nm (green) and 800 nm (red), respectively. The analyses of these images are noted above.

### Identification of proteins by mass spectrometry

Proteins were separated by SDS-PAGE (4–15% gradient gel; Bio-Rad). After staining with Sypro Ruby, the gel bands at 127 kDa, 97 kDa, 53 kDa, 42 kDa, 32 kDa, and 19 kDa were sliced for liquid chromatography–electrospray ionization–tandem mass spectrometry (LC–ESI–MS/MS). The proteins were digested in-gel with sequencing-grade trypsin, and the peptides were eluted and separated by in line C18 chromatography, as previously described [25]. In brief, the amino acid sequences of the peptide ions were obtained with an LTQ Orbitrap mass spectrometer (Thermo Finnigan). Peptides were detected, isolated, and fragmented to produce a tandem mass spectrum of specific fragment ions for each peptide. Peptide sequences were determined by the software program, Sequest (Thermo Fisher Scientific). All databases include a reversed version of all the sequences, and the data was filtered to between a one and two percent peptide false discovery rate (Table 2).

### Statistics

Two-way ANOVA was used to compare interactions between MyBPC paralogs (fMyBP-C and sMyBP-C) and age (adult and old) followed by Sidak’s multiple comparisons post-hoc analysis (GraphPad Prism 9, version 9.3.1). Student *T*-test was used to estimate differences between age groups for cMyBP-C relative content, DNP-s/fMyBP-C ratio, and DNP-cMyBP-C relative content. Values are expressed as means ± SEM. *P* ≤ 0.05 is accepted as significant.

## Results

### Identification and quantification of MyBP-C paralogs, myosin heavy chain, and actin in fast tibialis anterior and heart muscles from adult and old mice

**Fig. 1:**
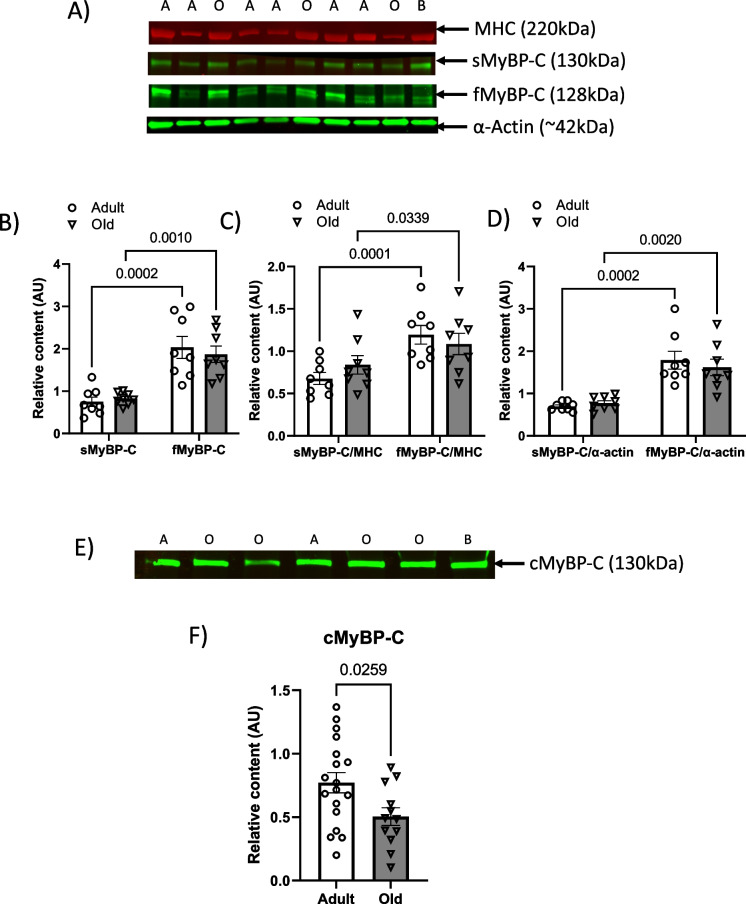
MHC, sMyBP-C, fMyBP-C, α-actin, and cMyBP-C protein expression in muscles from adult and old mice. **A** Representative Western blots for MHC, sMyBP-C, fMyBP-C, and α-actin in fast tibialis anterior muscles from adult (13 months) and old (29 months) mice. “A” indicates samples from adult mice and “O” indicates samples from old mice. “B” is the blot control sample. **B** The quantification of protein expression for the sMyBP-C and fMyBP-C paralogs in the fast tibialis anterior muscles from adult (open circle) and old (open triangle) mice. **C** Quantitative analysis of the expression of sMyBP-C/MHC and fMyBP-C/MHC in the tibialis anterior muscles from adult (open circle) and old (open triangle) mice. **D** Quantitative analysis of the expression of sMyBP-C/α-actin and fMyBP-C/α-actin in the tibialis anterior muscles from adult (open circle) and old (open triangle) mice. **E** Representative Western blot for cMyBP-C in cardiac left ventricles from adult (13 months) and old (29 months) mice. “A” indicates samples from adult mice and “O” indicates samples from old mice. “B” is the blot control sample. **F** The quantification of protein expression for the cMyBP-C in the left ventricles from adult (open circle) and old (open triangle) mice. Error bars represent ± SEM. Statistical analyses were performed by GraphPad Prism 9, version 9.3.1. AU arbitrary units, sMyBP-C slow MyBP-C paralog, fMyBP-C fast MyBP-C paralog, cMyBP-C cardiac MyBP-C paralog, MHC myosin heavy chain

To gain insight into the overall expression profile of the specific MyBP-C paralogs present in the fast TA muscles from adult and old mice, we analyzed the relative content of both the sMyBP-C (slow paralog) and fMyBP-C (fast paralog). We found both paralogs, sMyBP-C and fMyBP-C, constitutively expressed in the fast TA muscles from adult and old mice. In comparison to the sMyBP-C paralog, the relative abundance of the fMyBP-C paralog was significantly greater in the TA muscles from both age groups (2.7-fold greater in adult mice, 2.3-fold greater in old mice, Fig. 1A and B). The differential expression of the paralogs (fMyBP-C and sMyBP-C) is also noted when expressing the data as a percentage of total MyBP-C. In the fast TA muscles from adult mice, the fMyBP-C represented 72.5% of the total MyBP-C and sMyBP-C represented 27.5% of the total MyBP-C. In the fast TA muscles from old mice, 68.5% of the total MyBP-C was fMyBP-C and 31.5% was sMyBP-C. With aging, the relative abundance of the distinct paralogs did not significantly change (Fig. 1B).

Because sarcomeric protein stoichiometry is tightly maintained, MyBP-C is reported to impact contractility [26–28], and we found both skeletal MyBP-C paralogs (fMyBP-C and sMyBP-C) expressed in the fast TA muscles, we further analyzed the relative content of each specific MyBP-C paralog to that of the fast myosin heavy chain isoforms (MHC) and to α-actin expressed in the TA muscles. Figure 1C and D highlights the analyses of fMyBP-C/MHC, sMyBP-C/MHC, fMyBP-C/α-actin, sMyBP-C/α-actin from adult and old mice. We observed consistent and significant higher levels of fMyBP-C/MHC and fMyBP-C/α-actin in fast TA muscles in both adult and old mice compared to sMyBP-C/MHC and sMyBP-C/α-actin. Lastly, because cardiac myosin binding protein-C (cMyBP-C) is an important modulator of cardiac output (ventricular diastolic function), we next determined the relative expression levels in hearts from adult and old mice. We found the expression levels of cMyBP-C was greater (~ 24%) in the adult compared to the old group (Fig. 1F).

### Characterization and evaluation of the phosphorylation levels of MyBP-C in fast tibialis anterior and heart muscles from adult and old mice

**Fig. 2:**
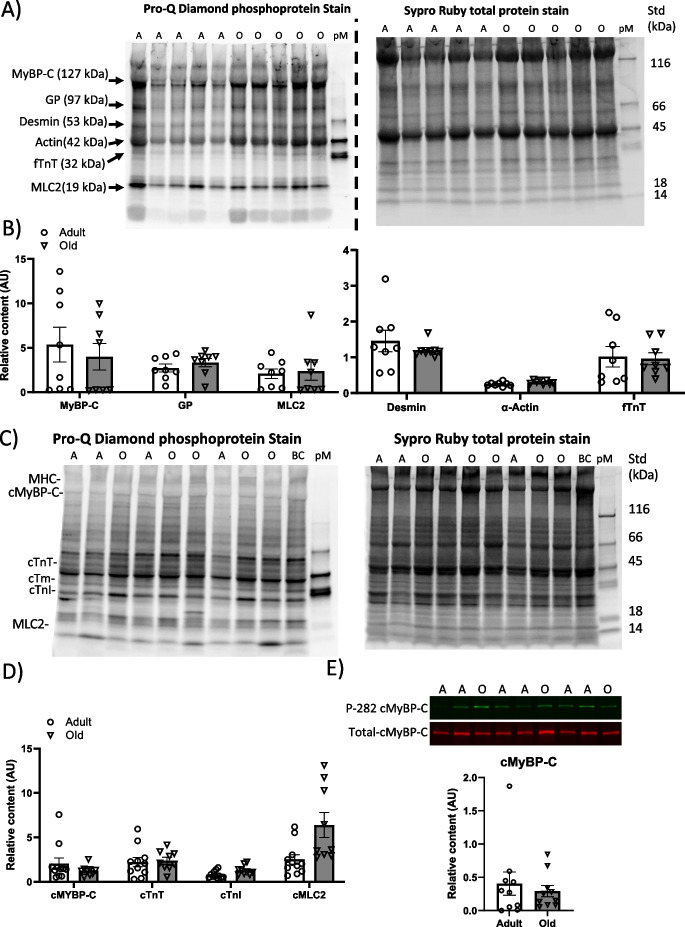
Phosphorylation levels of sarcomeric proteins in muscles from adult and old mice. **A** Representative Pro-Q Diamond staining of phosphorylated proteins in TA muscles on SDS gel electrophoresis that were extracted from adult (A, 13 months) and old (O, 29 months) mice. Subsequent Sypro Ruby staining of total proteins on the same gel. **B** Quantification of phosphorylation of MyBP-C (both sMyBP-C and fMyBP-C), PG, MLC2, desmin, actin, and fTnT (proteins confirmed by mass spectrometry) from Pro-Q-stained over the total specific protein (P/T ratio) in the Sypro Ruby-stained gels from adult (open circle) and old (open triangle) mice. **C** Representative Pro-Q Diamond staining of phosphorylated proteins in cardiac left ventricle muscles on SDS gel electrophoresis that were extracted from adult (A, 13 months) and old (O, 29 months) mice. Subsequent Sypro Ruby staining of total proteins on the same gel. **D** Quantification of phosphorylation of cMyBP-C, cTnT, cTnI, and cMLC2 from Pro-Q-stained over the total specific protein (P/T ratio) in the Sypro Ruby-stained gels. **E** Representative Western blot of cMyBP-C phosphorylated at S282 and total cMyBP-C in left ventricle muscles from adult (A) and old (O) mice. Quantification of cMyBP-C phosphorylation at S282 over the total cMyBP-C (P/T ratio) from adult (open circle) and old (open triangle) mice. Error bars represent ± SEM. Statistical analyses were performed by GraphPad Prism 9, version 9.3.1. AU arbitrary units (P/T ratio)

To determine the phosphorylation profile of MyBP-C (fMyBP-C and sMyBP-C) in the fast TA muscle from adult and old mice, we used standard SDS-PAGE along with Pro-Q Diamond phosphoprotein staining, a Sypro Ruby staining for protein amount, and mass spectrometry to confirm protein identities (Fig. 2A). Analysis indicated that the fast TA muscles from both adult and old contained phosphorylated MyBP-C proteins; however, the level of the phosphorylation (P/T ratio) did not differ between the two age groups for MyBP-C (Fig. 2B). Figure 2A shows the representative staining of Pro-Q Diamond phosphoprotein staining and Sypro Ruby staining for 10 skeletal muscle samples. In addition, mass spectrometry identified other phosphorylated myofilament proteins: glycogen phosphorylase, desmin, actin, troponin T, and myosin regulatory light chain 2. Although these MS-identified proteins show differential levels of phosphorylation (P/T ratio), the levels of phosphorylation for each protein did not differ with age (Fig. 2B).

Lastly, because cMyBP-C phosphorylation is important in regulating cardiac contractile function, we used Pro-Q Diamond phosphoprotein staining and the phospho-specific S282P antibody to evaluate the cMyBP-C Ser^282^ phosphorylation profile in the heart tissue from adult and old mice. Using both experimental techniques to detect phosphorylated cMyBP-C, the phosphorylation profile (MyBP-C and cMyBP-C Ser^282^ phosphorylation) did not change with age (Fig. 2C and E). Similar to the TA muscle, cardiac troponin (troponin T, troponin I) and myosin regulatory light chain 2 show differential levels of phosphorylation (P/T ratio) (Fig. 2D) and no difference with aging.

### Identification of carbonyl immunoreactivity in MyBP-C in fast tibialis anterior and heart muscles from adult and old mice

**Fig. 3:**
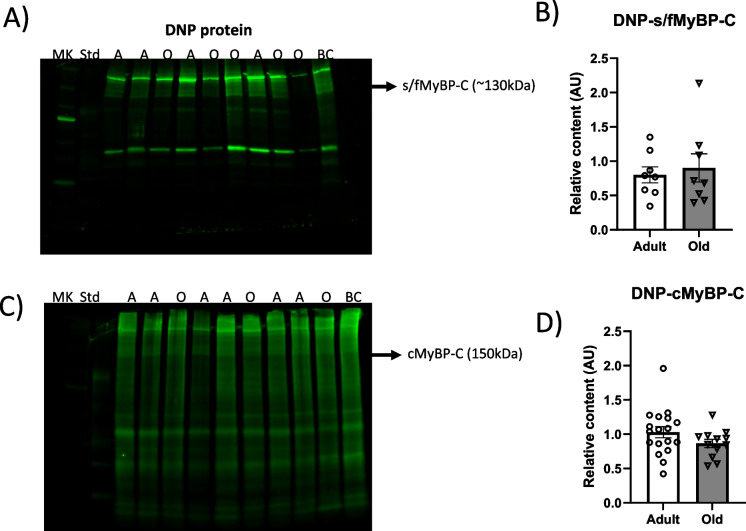
Carbonylated protein content in muscles from adult and old mice. **A** Representative OxyBlot (anti-DNP) to determine carbonylated content of s/fMyBP-C in tibialis anterior muscles from adult (A, 13 months) and old (O, 29 months) mice. **B** Quantification of anti-DNP s/fMyBP-C over the total protein content of MyBP-C from adult and old mice. **C** Representative OxyBlot to determine carbonylated content of cMyBP-C in left ventricles from adult (A, 13 months) and old (29 months) mice. **D** Quantification of anti-DNP cMyBP-C over the total protein of cMyBPC from adult and old mice. Error bars represent ± SEM. Statistical analyses were performed by GraphPad Prism 9, version 9.3.1. AU arbitrary units

To determine MyBP-C protein-bound carbonyls in fast TA and cardiac muscles from adult and old mice, we used immunostaining (anti-DNP, anti-fMyBP-C, anti-sMyBP-C, and anti-cMyBP-C). Anti-DNP immunostaining was detected in muscles from both adult and old mice, with prominent protein carbonyl immunoreactivities at ~ 130 kDa (TA muscle) and ~ 150 kDa (cardiac muscle), which were identified as fMyBP-C/sMyBP-C and cMyBP-C, respectively (Fig. 3A and C). The degree of MyBP-C protein-bound carbonyls in the fast TA muscles did not change between age groups (Fig. 3B). Similar to the fast TA muscles, the degree of cMyBP-C protein-bound carbonyls in the heart tissue did not differ between age groups (Fig. 3D).

## Discussion

Skeletal muscle dysfunction associated with aging involves a multitude of changes in muscle structure and function, including the sarcomeric proteins. Thus, the overall goal of this study was to begin to characterize MyBP-C and its paralogs in the fast TA muscle from adult and old mice. The main findings are as follows: First, both paralogs, sMyBP-C and fMyBP-C, are constitutively and differentially expressed in the fast TA muscles with the fMyBP-C paralog being predominant. Second, the composition profiles of the MyBP-C paralogs within the fast TA muscles are similar in adult and old mice. Third, the MyBP-C paralogs in fast skeletal and cardiac muscle are highly phosphorylated and oxidatively modified. Fourth, age-associated changes in the overall phosphorylation and carbonylation levels in MyBP-C from the TA and cardiac muscles are not observed, whereas an age-related reduction in the expression of the cMyBP-C paralog in cardiac muscle is observed.

### MyBP-C paralogs

In the current study, we found that both the sMyBP-C and the fMyBP-C paralogs are differentially expressed in adult (13 months) and old mice (29 months), with the fMyBP-C paralog being the most abundant paralog in the skeletal fast TA muscles, expanding our current understanding of MyBP-C composition within fast skeletal muscles. Differential expression of the MyBP-C paralogs in skeletal muscle is reported in the fast EDL muscle of adult rodents (rat and mouse) [8, 9, 26] and in the zebrafish experimental model [6]. Although the sMyBP-C paralog is detected in fast skeletal muscles investigated to date, the fMyBP-C paralog is in greater abundance in skeletal muscles characterized as fast-twitch muscles; whereas muscles characterized as slow-twitch such as the soleus muscle express only the sMyBP-C paralog [8, 26]. In a recent investigation using immunofluorescent-labeled MyBP-C in cryo-sectioned EDL muscles and imaged with confocal microscopy, the two MyBP-C paralogs showed specific spatial distribution within the cross-bridge-bearing C-zone of the sarcomere [26]. Indeed, our results do demonstrate clearly consistent and higher levels of fMyBP-C/MHC and fMyBP-C/α-actin compared to sMyBP-C/MHC and sMyBP-C/α-actin suggesting the possibility of specific spatial distributions of these two paralogs within the sarcomeres in the fast TA muscle; however, future investigations are required. Lastly, our results confirm that cMyBP-C paralog is exclusively expressed in the cardiac ventricles from adult and old mice [8].

There is a body of work incorporating comprehensive, detailed analyses of protein isoform composition in muscles and in single fibers highlighting age-related shifts in myosin heavy chains and myosin light chain isoforms that are both muscle and fiber-type dependent [4, 29]. For example, there is an age-related decrease in the relative content of myosin light chain 3f (MLC3f) in single MHC type II skeletal muscle fibers [30, 31]. Importantly, the reduction in MLC3f content is correlated with a decline in contraction velocity [30], which is restored by increasing the levels of MLC3f [30]. Thus, shifts in skeletal muscle protein isoform profiles impact contractile function and provide vital information regarding mechanisms contributing to the complex nature of sarcopenia. In fact, recent studies begin to address the biophysical functions of the two MyBP-C paralogs within skeletal muscles, providing a premise for the specific MyBP-C paralogs as players in sarcopenia [6, 7, 32]. Support for this idea is derived by two studies. First, ablation of fMyBP-C (genetic deletion) reveals this paralog is essential for contractility (force and speed of contraction), sarcomere integrity, and calcium sensitivity in the fast EDL muscle [32]. Further, these mice lacking fMyBP-C show impaired grip strength and increased susceptibility to muscle damage under a condition of chronic overload [32]. Second, reducing the levels of the sMyBP-C paralog (> 70%) in the mouse flexor digitorum brevis (FDB) and lumbrical muscles also results in altered thick filament organization, impaired force production, and altered contractile kinetics [7]. Collectively, these biophysical studies focused on the contractile functions of the specific MyBP-C paralogs imply MyBP-C is critical for generating maximal force. Therefore, in the current study, we were interested in investigating MyBP-C paralogs in the TA muscle where an age-associated decline in strength is found to increase the risk of falls in humans [33], neuromuscular dysfunctions are present in rodents and humans (e.g., [34]), and fast-twitch muscles are very susceptible to the aging process [35]. Next, we selected two age groups, 13 months and 29 months, representing two time points across the lifespan (100% and < 50% survival rates) for male C57Bl/6 mice and time points at which there are evidences of frailty and sarcopenia [24, 36]. The maintenance of the relative abundance levels of the two MyBP-C paralogs between 13 and 29 months of age in the current study suggests there is not a loss in the coordinated expression levels of the MyBP-C isoforms within the fast TA muscles. Indeed, the fMyBP-C paralog continues to be the predominant form, which likely contributes to the force-generating capacity in the TA muscles in old age. Interestingly, there is a reported reduction of the sMyBP-C paralog in the FDB muscles isolated from mice at earlier time points across the lifespan of the mouse, 2 and 14 months old [10]. It is important to note that the FDB muscle is characterized as a fast-twitch muscle, but it has an intermediate contractile phenotype compared to the fast contractile phenotype of the TA muscle [37]. Further, both muscles have a mixed fiber-type composition; yet, the FDB is composed of MHC type IIx (52%), IIa (44%), and I (4%) compared to the TA muscle with 5% type IIa, 35% type IIx, and 60% type IIb fibers [38, 39]. At this time, due to the dearth of reported studies focused on aging investigating the skeletal muscle MyBP-C paralogs, it is not possible to identify whether the findings are associated with the comparison between different age groups investigated (e.g., temporal progression of age across the lifespan 2–4 months vs 13 months as adult group) and/or the different muscles with varying fiber-type compositions and anatomical functions, the cross-sectional research design (investigating “successful agers”), etc. [34].

In contrast to healthy aging, the expression levels of the MyBP-C paralogs (both sMyBP-C and fMyBP-C) are markedly reduced in the dystrophic fast TA muscle from *mdx* mice suggesting the paralogs are regulated in the presence of physiological stressors [9]. Yet, the reported results from the limited number of studies characterizing the disease-induced alterations in the expression profiles of the MyBP-C paralogs are not easily explained at this time. First, the studies focus on the sMyBP-C paralog from the FDB muscle [9] resulting with limited information regarding the predominant fMyBP-C paralog from fast muscles. Second, the findings are not consistent in the presence of muscular dystrophy whereby there is a reduction in the expression levels of the sMyBP-C paralog and evidence of no change in expression levels from the same group of investigators [9, 10]. In the heart, our results report a decrease in the relative abundance of the cMyBP-C with aging, which to our knowledge has not been reported before. Collectively, the emerging results suggest that under stressful conditions, there may be a loss in the coordinated expression of the MyBP-C paralogs, which is muscle specific. Further, a loss in the coordinated expression of the sarcomeric proteins may play a role in muscle dysfunction with aging.

### Post-translational modifications: phosphorylative and oxidative

Interest in the role of skeletal MyBP-C phosphorylation and its impact on contractility is growing [40]. The initial biophysical studies focus on the sMyBP-C paralog and elegantly show that phosphorylation of sMyBP-C contributes to normal physiological function and by regulating cross-bridge recruitment, cross-bridge cycling kinetics, and internal drag forces, which oppose force generation, velocity of contraction, and overall muscle power output [40]. Yet, to our knowledge, relatively little is known about the impact of phosphorylation of the fMyBP-C paralog and its contractility. And similar to studies investigating cMyBP-C and aging discussed below, less work investigates the phosphorylation state of sMyBP-C and fMyBP-C paralogs with aging or disease in skeletal muscles. More broadly, the results from the current study and the limited published studies raise the possibility surrounding the complexity of the phosphorylation profiles in the skeletal muscle-specific MyBP-C paralogs. Our results, investigating age-associated alterations in phosphorylation levels in the sMyBP-C paralog and in the fMyBP-C, reveal no change in phosphorylation levels in the fast TA muscles from adult and old mice. Our data, reporting no change in sMyBP-C phosphorylation with age, aligns well with the reported no change in phosphorylation levels of sMyBP-C in the fast FDB muscles with age [10, 20] and expands our knowledge of the fMyBP-C paralog. The overall interpretation of these findings suggests that both MyBP-C paralogs are constitutively phosphorylated at basal levels and it seems these levels are maintained with aging in these two fast muscles investigated to date. In contrast, the phosphorylation state of sMyBP-C with disease is not as clear based in investigations using the *mdx* dystrophic mouse model whereby both an overall increase and an overall decrease [10] in skeletal sMyBP-C phosphorylation are reported from the same group of investigations [9]. At present, given the incomplete analyses of site-specific phosphorylation of key residues, we cannot rule out the impact of a shift in the phosphorylation profile as a contributing factor in muscle dysfunction with aging.

cMyBP-C phosphorylation regulates cardiac muscle contractility by several mechanisms including the number of cross-bridges strongly bound to actin and accelerating the kinetics of myosin-actin [41, 42]. The importance of cMyBP-C phosphorylation is clearly acknowledged whereby a reduction in cMyBP-C phosphorylation is associated with cardiac diseases (e.g., heart failure, atrial fibrillation) [43, 44] and with the development of cardiac dysfunction [45]. Site-specific phosphorylation of cMyBP-C is also very important because Ser282 phosphorylation is critical for subsequent phosphorylation of Ser302 [46] and phosphor-ablation of Ser282 impairs baseline diastolic function [46]. At this time, the limited number of studies investigating the phosphorylation status in cMyBP-C with aging report hypophosphorylation cMyBP-C at Ser282 in 28-month-old mice compared to 7-month-old mice (male C57Bl/6, *n* = 5–6) [19] and in 18–24-month-old mice compared to 2–6-month-old mice (grouped male and female genetically modified cMyBP-C(tWT), *n* = 5–6) [45]. In contrast, there is a report of no change in phosphorylation cMyBP-C at Ser307 in 24-month-old mice compared to 6-month-old mice [47]. Interestingly, site-specific phosphorylation levels (cMyBP-C Ser273 and Ser282) decreased in a linear manner with increasing age, whereas this correlation was not observed with cMyBP-C 302 phosphorylation in the genetically modified cMyBP-C(tWT) mice [45]. It is important to note that the expression levels of cMyBP-C in the genetically modified cMyBP-C(tWT) mice are not at 100% (at 72%); thus, the results may be related to the mouse strain and with the grouping of the male and female mice together [45]. In the current study investigating cardiac tissue from two C57Bl/6 male age groups (13 months and > 29 months, *n* = 10), we did observe phosphorylation of cMyBP-C at Ser 282; however, no reduction in the phosphorylated state at this specific residue was noted, even though we report a decrease in abundance of cMyBP-C (noted above). While our results do not report the hypophosphorylation of cMyBP-C at Ser282 as reported in mice comparing ages of 7 months and 18–24 months, the specific phosphorylatable sites in cMyBP-C show varied responses to aging as noted above [45, 47]. Given the evidence that implicates phosphorylation as a regulator of cardiac function, future studies focused on both contractility and characterization of the multiple specific sites of phosphorylation will provide valuable information [48].

Protein phosphorylation is known to modify contractile function in both skeletal and ventricular muscles. In this investigation, we used the common phosphorylation stain (Diamond Pro-Q), a total protein stain (Sypro), and mass spectrometry to confirm the identification of MyBP-C, to identify other phosphorylated proteins, and to determine whether aging impacts the basal phosphorylation levels in the fast TA and ventricular muscles. The findings are in line with the literature of readily phosphorylated myofilament proteins in ventricular and skeletal muscles and also the quantification levels in protein phosphorylation [45, 49–51]. Indeed, phosphorylation of troponin T, troponin I, MLC2, and GP modulates protein stability, myofilament Ca^2+^ sensitivity, contractility, and is involved in metabolic pathways, respectively [52–55]. There is evidence of changes in myofilament site-specific protein phosphorylation status in response to stress conditions (e.g., exercise, heart failure, diabetes) [56, 57], and the cellular biophysical studies demonstrate a strong link between site-specific residue phosphorylation and cardiac contractile function or dysfunction, with less work focused on skeletal fast muscles. In fact, profiles of site-specific phosphorylation within these identified cardiac myofilament proteins (e.g., cTnI) are associated with dysfunction [1]. We did not observe a global or overall change in the phosphorylation profile of these specific myofilament proteins with aging, which may be associated with the ages and the health status of the mice. Changes in site-specific residue phosphorylation in these skeletal muscle myofilament proteins with aging remain to be addressed.

The muscle is vulnerable to oxidative stress, and protein carbonylation is one of the most widely investigated and aging-relevant forms of oxidative stress-induced protein oxidation [3]. Protein carbonylation is irreversible, influences protein stability, and marks proteins for proteasomal degradation [58]. In particular, with age, fast-twitch muscles have about two times more proteins susceptible to carbonylation, with over 20 of these proteins showing significant increases in carbonylation compared to slow-twitch muscle [59]. Advanced bioinformatic analysis noted the carbonylated proteins belong to pathways and functional classes known to be impaired in aging skeletal muscle (e.g., cellular function/maintenance and cell death) [59]. There are causal relationships between enhanced protein carbonylation, the reduced content of the protein, and enhanced proteolysis in both skeletal and cardiac muscles with chronic disease such as COPD or in the presence of cardiotoxicity [60, 61], raising the possibility as a contributing factor in loss of protein function. Because of the emerging studies that provide insight into the multiple roles of MyBP-C paralogs in sarcomere structure/function and the evidence of oxidative stress in the skeletal muscle with aging, we sought to determine the carbonylation status of MyBP-C. Our investigation finds that under basal conditions, the MyBP-C paralogs exhibit protein carbonylation, which do not show an increase with age in the fast TA and cardiac muscles. We conclude from these findings that overall protein carbonylation of MyBP-C may not directly contribute to age-associated muscle dysfunction; however, functional implications were not assessed. In addition, MyBP-C protein is susceptible to protein degradation [58, 61, 62], which subsequently opposes an increase in carbonylated MyBP-C. While our studies focused on carbonylation, there are other post-translational modifications in MyBP-C that require investigation such as acetylation, glutathionylation, ubiquitination, citrullination, and O-GlcNAcylation that may affect sarcomeric structure and contractility [48]. For instance, there is a demonstrated relationship between glutathionylation of cMyBP-C and diastolic dysfunction [63].

## Conclusion

To date, not much is known about the skeletal MyBP-C paralogs in the fast TA muscles and whether their expression levels and post-translational modifications are affected by aging. This study demonstrates differential expression levels of sMyBP-C and fMyBP-C in the TA muscle, which are not affected by aging. The fast TA muscles from adult and old mice are composed mainly by the fMyBP-C paralog. Besides being highly phosphorylated, MyBP-C is carbonylated and these two post-translational modifications are not influenced by aging. In conclusion, our data emphasize and support the potential for MyBP-C in maintaining contractile function with aging. At this time, we cannot rule out other age-driven modifications nor the role of site-specific post-translational modifications in the MyBP-C paralogs; thus, future studies are needed.
